# Reducing lesion incidence in pork carcasses by heating foot-and-mouth disease vaccine before injection

**DOI:** 10.5713/ajas.19.0237

**Published:** 2019-08-03

**Authors:** Jaesung Cho, Eun Young Ko, Kyung Jo, Seonmin Lee, Sungbong Jang, Minho Song, Samooel Jung

**Affiliations:** 1 Division of Animal and Dairy Science, Chungnam National University, Daejeon 34134, Korea; 2 Dodram Pig Farmers’ Cooperative, Icheon 17405, Korea

**Keywords:** Foot-and Mouth Disease Vaccine, Pork, Lesion, Heat Treatment

## Abstract

**Objective:**

This study was conducted to investigate the effect of heating of foot-and-mouth disease (FMD) vaccine before injection, on the incidence of lesions at the injection site (pork butt), amount of discarded meat, and economical benefit.

**Methods:**

In total, 101,086 piglets raised in 30 farms, were vaccinated in the neck with 2 mL of FMD vaccine at 56 d and 84 d of age using a commercial syringe. The heat treatment group (48,511 pigs) was injected with the FMD vaccine after it had been heated in a water bath at 40°C for 20 min. After slaughter, the incidence of lesions on the pork butt was inspected, and the subsequent amount of discarded meat was recorded.

**Results:**

Heat treatment of FMD vaccine reduced the incident rate of lesions on the pork butt (p<0.01). Overall, 17.81% of the pigs in the heat treatment group had lesions, while the incident rate in the control group was 21.70%. The amount of discarded meat per head of total pigs and per head of pigs with lesions were significantly lower in the heat treatment group than the control group (p<0.01). Thus, the proportion of discarded meat to dressed carcass was lower in the heat treatment group (0.249%) compared with the control group (0.338%) (p<0.01). Farms that rear 1,000 sows can gain 1,863,289 KRW (1,600 USD) in one year when they adopt heat treatment of FMD vaccine before injection.

**Conclusion:**

Heat treatment of FMD vaccine using simple heat equipment (water bath) can be effective in reducing lesions caused by FMD vaccination and increase the economic benefits in pig farms.

## INTRODUCTION

An outbreak of infectious diseases in animal husbandry has resulted in great economic losses. Foot-and-mouth disease (FMD) is a highly contagious disease of cloven-hoofed animals and it is difficult to control this disease [1]. Vaccine injection is the most effective mode of preventing infectious disease outbreaks [2]. In South Korea, intensive vaccination campaigns for FMD began in 2011 because of a large outbreak of FMD in 2010 [3].

Although vaccination has contributed to controlling FMD, adverse reactions to the FMD vaccine in animals have been reported [4–6]. One of the adverse reactions of FMD vaccination is the incidence of lesions at the vaccination site. Incidence of lesions, such as abscess, fibrosis, and granuloma in animals, have been found at the vaccination sites of various vaccines, including the FMD vaccine [6–9]. The incidence of muscular lesions at the vaccination site in animals results in economic loss [3,9]. Ko et al [7] reported that the amount of abnormal meat in pork carcasses was dramatically increased by the incidence of lesions at the injection site after FMD vaccination. The abnormal meat that has lesions is discarded or trimmed. In pork, the lesions are mostly formed in the pork butt because the major site of FMD vaccination is the neck of an animal [8]. Pork butt is relatively expensive compared with other pork cuts, such as loin and ham, because it is preferable in South Korea, thus the incidence of lesions in pork butt results in high economic loss. Therefore, a method to reduce the incidence of lesions at the vaccination site of the FMD vaccine is required.

The lesion incidence at the vaccination site in animals is known to occur due to tissue damage and contamination caused by the needle [8]. Recent studies reported that the use of needle-free injection devises instead of needle syringes for vaccination of pig resulted in the increase of pork quality by reduction of muscular lesions with the inhibition of muscle damage [10,11]. However, the adaptation of needle-free injection devises in farms is costly because of the expensive devise, training of a worker, and maintenance [10].

Adjuvants are one of reasons for lesion incidence at vacci nation site of animals. It is added to vaccines to improve the immune response. However, adjuvants can generate lesions at the vaccination site by causing local reactions after intramuscular inoculation regardless of adjuvant types [9,12]. An oil adjuvant especially multiphasic emulsions (water in oil in water) is generally used in the FMD vaccine because of its effective short- and long-term immune response [3,13–15]. The incidence of lesions associated with local reactions, such as cell death and granulomatous inflammatory responses at the injection site, have been reported after vaccination with oil emulsion adjuvants [16–18]. The local reactions with oil adjuvant in muscle tissue could be caused by various factors including the toxicity of oil components and accumulation of vaccine in muscle tissue with low absorption rate [12,17]. Previous study [12] found a decrease in lesions as well as mortality of birds at the vaccination site, when the vaccine with an oil adjuvant was heated before injection, to increase the vaccine temperature that matches the body temperature of birds. Therefore, we hypothesized that heating of the FMD vaccine before injection would decrease the incidence of lesions at the injection site of the pig. Therefore, the aim of this study was to investigate the effect of heating the FMD vaccine before injection to pigs, on the incidence of lesions on the pork butt, amount of discarded meat, and economic benefit.

## MATERIALS AND METHODS

### Animals and treatments

This study used a total of 101,086 pigs. Pigs were raised in 30 farms and cared for in accordance with the guidelines of the Dodram Pig Farmers’ Cooperative. The piglets were vaccinated two times, in the left neck, with 2 mL of the FMD vaccine (Aftopor, Merial Amimal Health Ltd., Surrey, UK), which was composed of serotypes O, A, and Asia 1 emulsified with the adjuvant Montanide ISA 206 (water-in-oil-in-water), by commercial syringe at 56 d and 84 d of age. Pigs were slaughtered for 8 months in a slaughter house of Dodram livestock processing complex (Anseong, Korea). The control group (52,575 pigs) was vaccinated with the FMD vaccine without heat treatment. The heat treatment group (48,511 pigs) was injected with the FMD vaccine after heating the vaccine in a water bath at 40°C for 20 min.

### Inspection of lesions on the Boston butt

The pork carcasses were deboned in Dodram livestock processing complex (Anseong, Korea). The incidence of lesions, such as abscess, fibrosis, and granuloma, on the pork butt was inspected by plant workers. The number of pork carcasses with lesions on the pork butt was recorded, along with the amount of discarded meat.

### Statistical analysis

The data were aligned according to the time (month), farm, number of pigs participating in the experiment, incidence rate of lesions, proportion of discarded meat, and information that indicates the use of heat treatment. The data were used to perform the equality tests of the means of the treated and untreated groups and to examine the effects of heating the FMD vaccine on the incidence rate of lesions on the pork butt and amount of discarded meat.

The t-test was used to perform the equality tests for the two types of means in the treated and untreated groups, including the mean of the incidence rate of lesions, mean of discarded meat/each carcass of total pork, mean of discarded meat/each carcass of pork having lesions, and mean of the proportion of discarded meat/each carcass of total pork. The null hypothesis was that the means of the two groups are not statistically different from each other.

The random- and fixed-effect models, in the form of the following linear equations, were estimated to examine the effects of heat treatment on the FMD vaccine.

Equation 1 $$Y_{it}=\alpha_{0}+\alpha_{1}X_{it}+\alpha_{2}K_{it}+v_{i}+e_{it}$$

Equation 2 $$Z_{it}=\beta_{0}+\beta_{1}X_{\text{it}}+\beta_{2}K_{it}+v_{i}+e_{it}$$

where *Y_it_* is the incidence rate of lesions, *Z_it_* is the proportion of discarded meat calculated by dividing the weight of the discarded meat by the carcass weight, *X_it_* is the dummy variable for indicating heat treatment, *K_it_* is the number of pigs, *v_i_* is the unit-specific error term, *e_it_* is the usual error term, *i* denotes the cross-section identifier, and *t* denotes the time identifier. *Y_it_* and *Z_it_* are expressed as percentages in the data.

The number of pigs is included in the model to consider the effects of the differences in the number of experiments across farms and time. The models were estimated using Stata 14 software [19]. The estimation results were compared to identify the most appropriate model for estimating the effects of heating the FMD vaccine on the incidence of lesions and proportion of discarded meat. In particular, the Hausman specification test was implemented to compare the fixed- and random-effect models [20].

### Economic analysis

The economic benefit of heating the FMD vaccine at the farm level is the increase in normal meat production by reducing the incidence rate of lesions and proportion of discarded meat. The revenue of a farm that does not heat the FMD vaccine can be represented by Equation 3.

Equation 3 $$B_{t}=(1-\gamma )R_{t}+\gamma R_{t}(1-\delta )$$

where *B_t_* is the total revenue per year of a farm that does not heat the FMD vaccine, *γ* is the incidence rate of lesions, *δ* is the proportion of wasted meat, and *R_t_* is the yearly revenue of the farm from selling pigs that have no lesions, which is calculated by the following equation.

Equation 4 $$R_{t}=SN_{t}SW_{t}P_{t}$$

where *R_t_* is the yearly revenue of the farm from selling pigs that have no lesions, *SN_t_* is the number of pigs slaughtered per year, *SW_t_* is the average carcass weight per slaughtered pig, and *P_t_* is the average price of pork.

On the other hand, the revenue of a farm that heats the FMD vaccine can be represented by Equation 5, which assumes that heating the FMD vaccine reduces the incidence rate of lesions from *γ* to *γ′* and proportion of discarded meat from *δ* to *δ′*.

Equation 5 $$B_{t}^{\prime }=(1-\gamma^{\prime })R_{t}+\gamma^{\prime }R_{t}(1-\delta^{\prime })$$

Therefore, the economic benefit, at the farm level, of heating the FMD vaccine can be calculated by subtracting Equation 3 from Equation 5, as below.

Equation 6 $$E_{t}=R_{t}(\gamma \delta -\gamma^{\prime }\delta^{\prime })$$

where *E_t_* is the economic benefit, at the farm level, of heating the FMD vaccine. *γ′* can be obtained by (*γ*+*α*_1_), using *α*_1_ from Equation 1, and *δ′* can be obtained by (*δ*+*β*_1_), using *β*_1_ from Equation 2 (The signs of *α*_1_ and *β*_1_ are expected to be minus).

Heating the FMD vaccine results in costs from purchas ing, maintaining, and operating the heating equipment. The purchasing cost of the heating equipment is a fixed cost. The maintaining and operating costs of the heating equipment are variable costs. With the fixed cost being *I* and variable costs being *V_t_*, the net present value (NPV) of a farm introducing heating equipment can be represented by the following equation.

Equation 7 $$NPV=\sum_{t=1}^{T}\left(\frac{E_{t}-V_{t}}{{\left(1+r\right)}^{t}}\right)-I$$

where NPV is the net present value, *r* is the annual interest rate, *T* is the duration of planning, *E_t_* is the annual economic benefit of heating the FMD vaccine in Equation 6,*V_t_* is the annual variable costs of heating the FMD vaccine, and *I* is the fixed cost of heating the FMD vaccine.

A summary of the independent variables in Equation 3 to 7 is presented in Table 1, with the exception of *γ′* and *δ′*, which are only known after estimating Equation 1 and 2.

## RESULTS AND DISCUSSION

**Figure 1:**
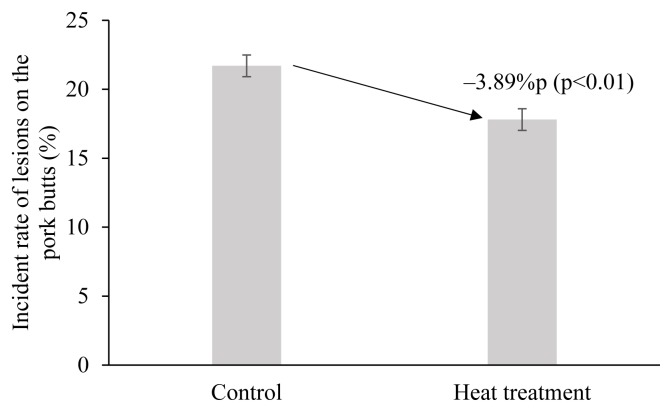
Incident rate of lesions at the injection site (pork butt) of foot-and-mouth disease vaccine after heat treatment at 40°C for 20 min. Error bars are standard error.

The incident rate of lesions on the pork butt (vaccination site) was 21.70% in the control group (Figure 1). This value is higher than 19.17% of a previous study [11]. In the present study, the FMD vaccine was injected in the neck, while the vaccination site was the ham in the previous study [11]. Ko et al [2] found that the incident rate of lesions differed with different sites of injection, and it was higher in the neck than in pork ham.

The estimation results of the random- and fixed-effect models from Equation 1 indicated that heat treatment has a statistically significant effect on the incidence of lesions (p<0.01). The adoption of heat treatment reduces the incidence of lesions by 3.89%p and 3.92%p in the random- and fixed-effect models, respectively. On the other hand, the effect that the number of pigs had on the incidence of lesions was almost none and not statistically significant. In addition, the Hausmann specification result shows that p>χ^2^ = 0.943, which implies that there should be no systematic difference between the two estimators. Therefore, a random-effect model is a more appropriate model for examining the effects of heat treatment on the incidence of lesions. The incident rate of lesions in the heat treatment group was 17.81%, which was a reduction of 3.89%p compared with the control group (Figure 1).

**Figure 2:**
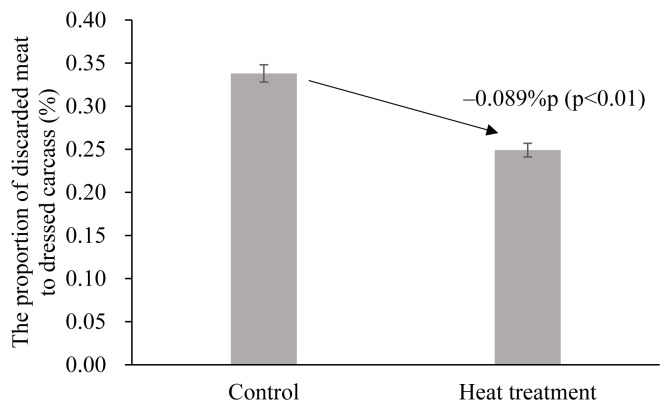
Proportion of discarded meat to dressed carcass (%) of pigs injected with foot-and-mouth disease vaccine after heat treatment at 40°C for 20 min. Error bars are standard error.

**Figure 3:**
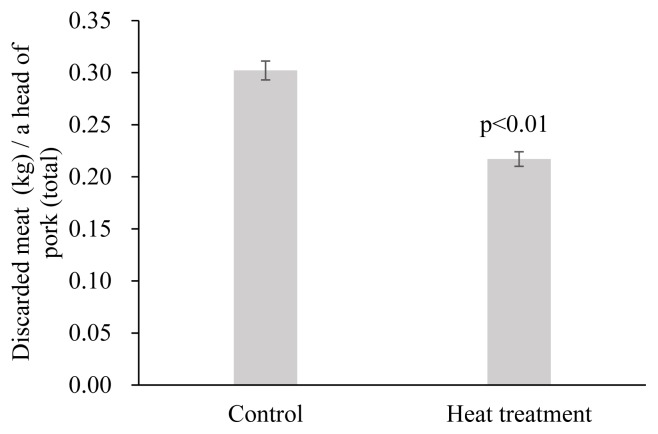
Amount of discarded meat (kg) per a head of total pigs injected with foot-and-mouth disease vaccine after heat treatment at 40°C for 20 min. Error bars are standard error.

**Figure 4:**
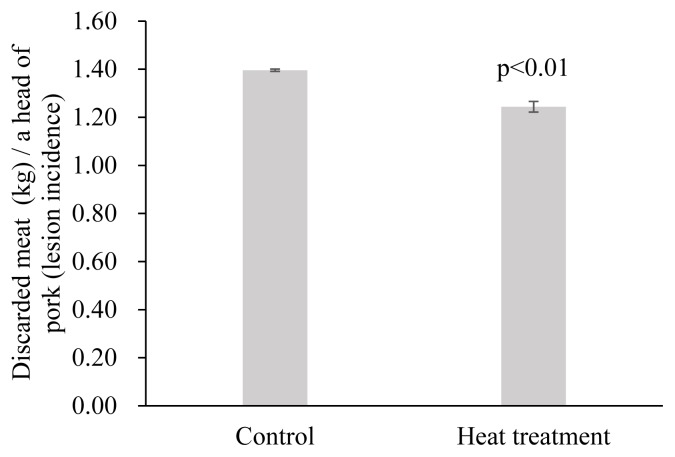
Amount of discarded meat (kg) per a head of pigs with lesions injected with foot-and-mouth disease vaccine after heat treatment at 40°C for 20 min. Error bars are standard error.

The estimation results of the random- and fixed-effect models from Equation 2 showed that heat treatment also has a statistically significant effect on the proportion of discarded meat to dressed carcass (p<0.01). The proportion of discarded meat to dressed carcass in the control group was 0.338% (Figure 2). Heat treatment of the FMD vaccine before injection reduced the proportion of discarded meat by 0.089%p and 0.090% in the random- and fixed-effect models, respectively. The random-effect model is a more appropriate model for examining the effects of heat treatment on the proportion of discarded meat to dressed carcass, because the Hausmann specification test result showed that p>χ^2^ = 0.875, which implies that there should be no systematic difference between the two estimators. From the results, heat treatment of the vaccine before injection resulted in 0.249% of the proportion of discarded meat to dressed carcass, by the reduction of 0.089%p compared with the control group (Figure 2). The reduced proportion of discarded meat in the heat treatment group was attributed to the reduction in the lesion incident rate and lesion size in the pork butts. The discarded meat in the pork butt per pork carcass of total pork was lower in the heat treatment group than the control group (Figure 3, p<0.01). In addition, the meat discarded from pork having lesions (discarded meat per a head of pigs with lesions) was 1.24 kg in the heat treatment group, which was statistically lower than the 1.40 kg in the control group (Figure 4, p<0.01).

The lesion incidence caused by the local reaction of adjuvant at the injection site is well known side effect of vaccination [9,13–15,17]. This side effects were found in poultry, mouse, rabbit, and cattle as well as pig [17,18]. The various factors such as toxicity of oil components and auto-allergic responses of oil adjuvants for the lesion incidence at the vaccination site of animals were reported [21,22]. Previous study reported that the use of high purity oil for the production of oil adjuvant decreased the local reaction at the injection site of vaccine [14,22]. In addition, the local reactions at the injection site of vaccines composed of an oil emulsion adjuvant was associated with the long-lasting deposit of oil emulsion adjuvant in the muscle tissue [17,18]. In the present study, the heating vaccine to the similar body temperature of the pigs had effects on the reduction of lesion incidence and discarded meat per a head of pigs with lesions. This result could be caused by the increase of vaccine absorption into muscle tissue. Few other studies have reported the effect of vaccine temperature on the lesion incidence in vaccinated animals. Burns et al [12] found that heating the oil emulsion vaccine to 41°C before injection, reduced the lesion incidence and size in chicken breast meat after vaccination. They reported that the reduction in the lesion incidence and size was caused by the rapid absorption of the vaccine into the muscle tissue [12].

The NPV is the summation of the present value of a se ries of cash flow over a certain planning duration. A value greater than zero implies that the investment is economically beneficial. The NPV in Equation 7 was calculated using the estimated coefficients of heat treatment, which were −3.893 and −0.089, and the result is shown in Table 2. The results indicate that the usage of heating equipment on the FMD vaccine generates a positive economic benefit for a farm with more than 100 sows, even if the farm uses the heating equipment for only one year. Farms that rear 1,000 sows can benefit from 1,863,289 KRW (1,600 USD), in one year, when they adopt heat treatment of the FMD vaccine before injection. Ko et al [11] reported that the use of a transdermal needle-free injection for FMD vaccination effectively reduced the incidence rate of lesions in pork carcasses from 19.17% to 4.35%. However, the needle-free injection requires a high [10]. Therefore, heat treatment of the FMD vaccine using simple heating equipment (water bath) can be effective in reducing the lesions caused by FMD vaccination and would be economically beneficial to pig farms.

## Figures and Tables

**Table 1:** Summary of the independent variables

Variable	Value	Unit	Source
*SN_t_*	1,780; 8,900; 17,800	head	Assumed, KREI 2019 [23]1)
*SW_t_*	89.36	kg/head	Based on the experimental data
*P_t_*	4,497	KRW/kg	MAFRA 2018 [24]
*γ*	21.66	%	Based on the experimental data
*δ*	0.34	%	Based on the experimental data
*V_t_*	0	KRW	Assumed2)
*I*	120,000	KRW	Market price3)
*r*	0.045	Rate	MOEF 2018 [25]
*T*	1; 5; 10	Year	Assumed

1) Marketed-pig per sow per year of 17.8 [23] and three types of hypothetical farm size are assumed: farms with 100, 500, and 1,000 sows.

2) Only negligible amounts of electricity and labor are required to maintain and operate the heating equipment for foot-and-mouth disease vaccine.

3) Market price is obtained by contacting the heating equipment supplier directly.

**Table 2:** Net present value of a farm introducing heating equipment

Planning duration	Sow numbers		

	100 sows	500 sows	1,000 sows
1	78,329	871,644	1,863,289
5	789,839	4,429,194	8,978,389
10	1,519,940	8,079,700	16,279,401

Unit is KRW.
